# On the Exchange of the Deciduous for the Permanent Incisor Teeth

**Published:** 1847-03

**Authors:** William Lintott


### ARTICLE VII.

### *On the Exchange of the Deciduous for the Permanent Incisor Teeth.* By WILLIAM LINTOTT, ESQ.

ONE of the most prolific sources of future mischief to the teeth, is the mal-practice perpetrated in the premature removal of deciduous teeth. Though strongly deprecating an excess of interference, I am by no means an advocate for leaving nature entirely to her own efforts. We have the power of modifyng considerably the natural process of the second dentition, retarding it by the adoption of proper means for the preservation of the deciduous teeth, and accelerating it by the removal of those, which, in consequence of the absorption of their fangs proceeding too slowly, become impediments to the eruption of their successors. In very many instances, by the judicious and timely application of mechanical force, we may prevent or remedy deformities, which, if neglected, not only become detrimental to personal appearance, but also eventually destroy the strongest teeth; but these measures should be adopted with the utmost caution.

It is frequently stated that the children of the poorer classes of society, escaping the interference of the dentist, have better teeth, with more regularity of arrangement, than those for whom his services are put in requisition. I cannot admit this to be the case in cities or large towns. In healthy situations in the country, there is undoubtedly less of irregularity and its consequences to be seen, but not to a greater extent than may be duly expected to result from simple constitutional causes, dependant on the more perfectly discharged functions of the animal economy. I do not intend in these observations to allude to the structural development of the teeth, as influenced by healthy, or morbid conditions of the system; but to their arrangement in the alveolar arch only.

In cases of ordinary health, the period at which the advice and assistance of the professed dentist becomes indispensably necessary for the child, is that when the four lower incisor teeth have completed their eruption from the gum. The average space of the alveolar border occupied by the four deciduous incisor teeth is ten-sixteenths of an inch, whilst their successors require from thirteen to fourteen-sixteenths. It is evident, then, that the permanent incisors at their first appearance must present themselves in an irregular and crowded manner. In these cases I have observed it to be the common practice to remove the deciduous canine teeth, and thus at once liberate the permanent incisors from the obstacle to their regular arrangement. This object I admit is effected, but at what cost, with what certainty of entailing upon the patient more inconvenient irregularity at a future period!

What is the relative state of growth and position of the bicuspids, and of the permanent canine teeth, at the time this operation is performed? The anterior bicuspids are the teeth that in due natural succession, should next emerge from the gum, and they will be found lying rather above and behind the permanent canines, pressing upon and promoting absorption of the fangs of the anterior deciduous molars, and inclining slightly forwards in the direction of the space which has been made by the extraction of the deciduous canines. Now if the effects of this operation could be limited to the liberation of the permanent incisors, the practice would not be objectionable, but unfortunately in the majority of cases so treated it will be found, that the growth of the anterior bicuspid teeth is thereby accelerated, but in a lateral direction, that the due absorption and dislodgment of the anterior temporary molars is consequently retarded, and in fine, that the space naturally destined for the permanent canine tooth is pre-occupied by the lateral incisor and the anterior bicuspid tooth, so that the canine must make its way through the external alveolar plate, and will most probably project beyond the arch of the superior maxilla. Thus is a formidable case of irregularity, involving probably the loss of the canine teeth, or at best, of the bicuspids, brought about by mal-practice, inasmuch as, if instead of removing the deciduous canines, the operator had extracted the anterior temporary molars, the desired arrangement of the incisor teeth would have been quite as surely attained, if not so expeditiously, the deciduous canines giving way before the pressure produced by the upward growth of the incisors, until checked by the advance of the anterior bicuspids in their proper position, and then remaining to secure space for their permanent successors.

The next exchange then made would be that of the posterior deciduous molars for the posterior bicuspids. The average space occupied by the bicuspids is nine-sixteenths of an inch, that before filled by the deciduous molars, twelve-sixteenths so that the incisor teeth thus gain a clear three-sixteenths, in addition to the increased space afforded by the growth of the maxillæ. The expansion of the alveolar border is considerably aided by the pressure of the teeth during their growth, and by the premature removal of any of the deciduous teeth, and especially of the canines, not only is the patient deprived of this advantage, but a tendency to contraction is induced. In my own practice, whenever I have observed that caries has commenced in the crowns of the deciduous molars, I have taken every opportunity afforded me, of filling the cavities with tin-foil; and this treatment has been attended with the best results. In addition to securing a due expansion of the maxillæ, the young patient escapes much pain, and the development of the permanent teeth proceeds uninfluenced by that morbid condition of surrounding structure resulting from the presence of carious teeth. The preservation of the crown of the deciduous tooth does not interfere with the natural absorption of the fangs, so that the eruption of the permanent teeth is not unduly retarded by this practice.

I have applied these remarks especially to the inferior maxilla, because I have observed it to be most frequently selected for mischievous interference, as well as because upon its contraction or due expansion, the form and width of the superior maxilla, and consequently the arrangment of the upper teeth, materially depends.

